# Study of Physico-Chemical Characteristics of Wastewater in an Urban Agglomeration in Romania

**DOI:** 10.1100/2012/549028

**Published:** 2012-08-01

**Authors:** Paula Popa, Mihaela Timofti, Mirela Voiculescu, Silvia Dragan, Catalin Trif, Lucian P. Georgescu

**Affiliations:** Department of Chemistry, Physics and Environment, European Centre of Excellence for the Environment, “Dunarea de Jos” University of Galati, Street Domneasca No. 111, 800201 Galati, Romania

## Abstract

This study investigates the level of wastewater pollution by analyzing its chemical characteristics at five wastewater collectors. Samples are collected before they discharge into the Danube during a monitoring campaign of two weeks. Organic and inorganic compounds, heavy metals, and biogenic compounds have been analyzed using potentiometric and spectrophotometric methods. Experimental results show that the quality of wastewater varies from site to site and it greatly depends on the origin of the wastewater. Correlation analysis was used in order to identify possible relationships between concentrations of various analyzed parameters, which could be used in selecting the appropriate method for wastewater treatment to be implemented at wastewater plants.

## 1. Introduction

Sources of wastewater in the selected area are microindustries (like laundries, hotels, hospitals, etc.), macroindustries (industrial wastewater) and household activities (domestic wastewater). Wastewater is collected through sewage systems (underground sewage pipes) to one or more centralized Sewage Treatment Plants (STPs), where, ideally, the sewage water is treated. However, in cities and towns with old sewage systems treatment stations sometimes simply do not exist or, if they exist, they might not be properly equipped for an efficient treatment. Even when all establishments are connected to the sewage system, the designed capacities are often exceeded, resulting in a less efficient sewage system and occasional leaks. 

Studies of water quality in various effluents [1–9] revealed that anthropogenic activities have an important negative impact on water quality in the downstream sections of the major rivers. This is a result of cumulative effects from upstream development but also from inadequate wastewater treatment facilities [10]. Water quality decay, characterized by important modifications of chemical oxygen demand (COD), total suspended solids (TSSs), total nitrogen (TN), total phosphorous (TP), iron (Fe), nickel (Ni), copper (Cu), zinc (Zn), lead (Pb), and so forth [11] are the result of wastewater discharge in rivers [10]. Water-related environmental quality has been shown to be far from adequate due to unknown characteristics of wastewater [12]. Thus an important element in preventing and controlling river pollution by an effective management of STP is the existence of reliable and accurate information about the concentrations of pollutants in wastewater. Studies of wastewater in Danube basins can be found, for instance, in central and eastern European countries [13–15], but we are not aware of extensive studies of wastewater quality at regional/national level in Romania. 

This paper analyses the chemical composition of wastewater at several collectors/stations in an important Romanian city, Galati, before being discharged into natural receptors, which in this case are the Danube and Siret Rivers. No sewage treatment existed when the monitoring campaign took place, except the mechanical separation. The study presented here is part of a larger project aiming at establishing the best treatment technology of wastewater at each station. Presently this project is in the implementation stage at all stations. Possible relationships between concentrations of various chemical residues in wastewater and with pollution sources are also investigated. The study is based on daily measurements of chemical parameters at five city collectors in Galati, Romania, during a two-week campaign in February 2010.

## 2. Experimental Analysis

### 2.1. Location of Sampling Sites

**Figure 1:**
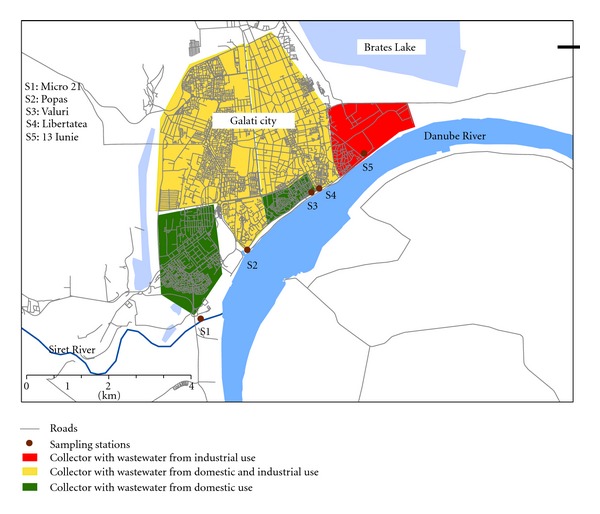
Monitoring sampling sites of wastewater from Galati city.

Galati-Braila area is the second urban agglomeration in Romania after Bucharest, which is located in Romania at the confluence of three major rivers: Danube, Siret, and Prut. The wastewater average flow is about 100000 m^3^/day [16]. The drainage system covers an area of 2300 ha, serving approximately 99% of the population (approximately 300000 habitants). The basic drainage system is very old, dating back to the end of the 19th century, and was extended along with the expansion of the city due to demographic and industrial evolution. There are several collectors that collect wastewater and rainwater from various areas with very different characteristics, according to the existing water-pipe drainage system. There is no treatment at any station, except for simple mechanical separation. However, industrial wastewater is pretreated before being discharged in the city system. The five wastewater collectors are denoted in the following as S1, S2,…, S5. Four of them discharge in the Danube River and the fifth discharges in the Siret River (which is an affluent of Danube River).  Figure 1 shows the distribution of the monitoring sites and highlights the type of collecting area (domestic, industrial, or mixed). For the sake of brevity, these stations will be named in the present paper as “domestic,” “mixed,” and “industrial” stations, according to the type of collected wastewater. The mixture between domestic and industrial water at the two mixed collectors is the result of changes in city planning and various transformations of small/medium enterprises. 

Technical details about each collector/station can be found in Table 1. The first station, S1, collects 10% of the total quantity of wastewater. A high percentage of the water collected at this station comes from domestic sources from the south part of the city (more than 96%). Station S2 collects 64% of the total daily flow of wastewater, out of which 30% comes from domestic sources and the rest (70%) is industrial. Most of the industrial sources in this area are food-production units (milk, braid, wine) while the domestic sources include 20 schools, 4 hospitals, and important social objectives. Station S3 is located in the old part of the city and collects 5% of the total wastewater and has domestic sources. At the fourth station, S4, 11% of the quantity of wastewater is collected from domestic (70%) and industrial (30%) sources. The last collector, S5, collects wastewater from the industrial area of the city, where the most important objectives are a shipyard, metallurgical, and mechanical plants and transport stations.

### 2.2. Physico-Chemical Parameters and Methods of Analysis

The physico-chemical parameters which were measured are the following:

- pH;
- chemical oxygen demand (COD) and dissolved oxygen (DO);
- nutrients such as nitrate (N-NO_3_) and phosphate (P-PO_4_) (these were included due to their impact on the eutrophication phenomenon);
- metals such as aluminum (Al^+3^), soluble iron (Fe^+2^), and cadmium (Cd^+2^).

The pH and DO were determined *in situ* using a portable multiparameter analyzer. Other chemical parameters such as COD, metals and nutrients were determined according to the standard analytical methods for the examination of water and wastewater [17].

The COD values reflect the organic and inorganic compounds oxidized by dichromate with the following exceptions: some heterocyclic compounds (e.g., pyridine), quaternary nitrogen compounds, and readily volatile hydrocarbons [18]. The concentration of metals (Al^+3^, Cd^+2^, Fe^+2^) was determined as a result of their toxicity. 

The value of pH was analyzed according to the Romanian Standard [19] using a portable multiparameter analyzer, Consort C932. 

COD parameter was measured using COD Vials (COD 25–1500 mg/L, Merck, Germany). The digestion process of 3 mL aliquots was carried out in the COD Vials for 2 h at 148°C. The absorbance level of the digested samples was then measured with a spectrophotometer at *λ* = 605 nm (Spectroquant NOVA 60, Merck, Germany), the method being analogous to EPA methods [20], US Standard Methods [17], and Romanian Standard Methods [21].

The DO parameter was analyzed according to Romanian Standard [22] using a portable multiparameter analyzer, Consort C932.

Aluminum ions (Al^+3^) were determined using Al Vials (Aluminum Test 0.020–1.20 mg/L, Merck, Germany) in a way analogous to US Standard Methods [17]. The absorbance levels of the samples were then measured with a spectrophotometer (Spectroquant NOVA 60; Merck, Germany) at *λ* = 550 nm. The method was based on reaction between aluminum ions and Chromazurol S, in weakly acidic-acetate buffered solution, to form a blue-violet compound that is determined spectrophotometrically. The pH of the sample must be within range 3–10. Whether necessary, the pH will be adjusted with sodium hydroxide solution or sulphuric acid. 

Iron concentration (Fe^+2^) was determined using Iron Vials (Iron Test 0.005–5.00 mg/L, Merck, Germany) and their absorbance levels were then measured using a spectrophotometer (Spectroquant NOVA 60; Merck, Germany) at *λ* = 565 nm. The method was based on reducing all iron ions (Fe^+3^) to iron ions (Fe^+2^). In a thioglycolate-buffered medium, these react with a triazine derivative to form a red-violet complex which is spectrophotometrically determined. The pH must be within range 3–11. Where necessary the pH was adjusted with sodium hydroxide solution or sulphuric acid. 

Cadmium ions (Cd^+2^) were determined using Cadmium Vials (Cadmium Test 0.005–5.00 mg/L, Merck, Germany), their absorbance levels being measured with a spectrophotometer (Spectroquant NOVA 60; Merck, Germany) at *λ* = 525 nm. The method was based on the reaction of cadmium ions with a cadion derivative (cadion-trivial name for 1-(4-nitrophenyl)-3-(4-phenylazophenyl)triazene), in alkaline solution, to form a red complex that is determined spectrophotometrically. The pH must be within the range 3–11, and, if not, the pH will be adjusted with sodium hydroxide solution or sulphuric acid. 

Nitrogen content was determined using Nitrate Vials (Nitrate Cell test in seawater 0.10–3.00 mg/L NO_3_-N or 0.4–13.3 mg/L NO_3_^−^, Merck, Germany). The method being based on the reaction of nitrate ions with resorcinol, in the presence of chloride, in a strongly sulphuric acid solution, to form a red-violet indophenols dye that is determined spectrophotometrically. The absorbance levels of the samples were then measured with a spectrophotometer (Spectroquant NOVA 60; Merck, Germany) at *λ* = 500 nm. 

Phosphorous content was determined using Phosphate Vials (Phosphate Cell Test 0.5–25.0 mg/L PO_4_-P or 1.5–76.7 mg/L PO_4_^−3^, Merck, Germany) with a method that was analogous to the US Standard Methods [17]. The method was based on the reaction of orthophosphate anions, in a sulphuric solution, with ammonium vanadate and ammonium heptamolybdate to form orange-yellow molybdo-vanado-phosphoric acid that is determined spectrophotometrically (“VM” method). The absorbance levels of the samples were then measured with a spectrophotometer (Spectroquant NOVA 60; Merck, Germany) at *λ* = 410 nm. 

All results were compared with standardized levels for wastewater quality found in accordance with European Commission Directive [23] and Romanian law [24]. 

## 3. Results and Discussion

### 3.1. The Acidity (pH)

**Figure 2:**
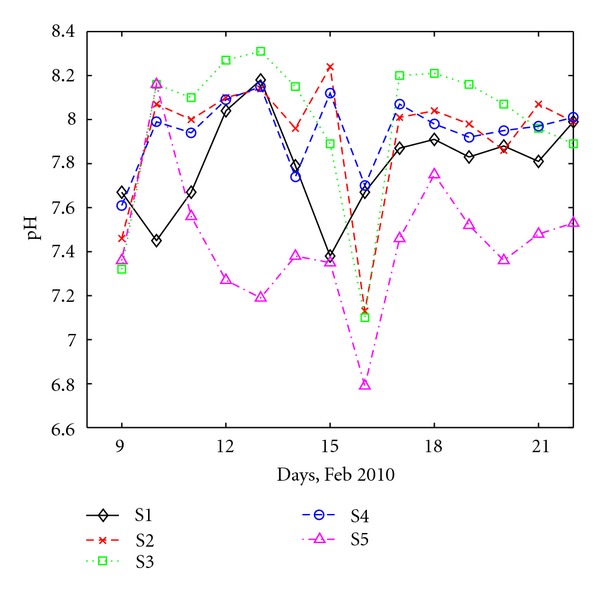
Daily variation of pH at all sites.

The results for pH for all the investigated five collectors are shown in Figure 2. 

 Generally, the wastewater collected at the monitored sites is slightly alkaline. The pH varies between 6.8 and 8.3—average value 7.82—thus the pH values are within the accepted range for Danube River according to the Romanian law [24], which is between 6.5 and 9.0. The pH variation is relatively similar at collectors S1–S4 (domestic and/or mixed domestic-industrial contribution). Lower pH values are observed at S5, which is dominated by industrial wastewater, originating from major enterprises and heavy industry. However, these values are not too low, since usually pH values for industrial wastewater are smaller than 6.5.

A significant decrease in the pH value was observed during the 8th day of the analyzed period at each station. Interestingly, a heavy snowfall took place at that particular time, thus the decrease could be attributed to the mixing between wastewater and a high quantity of low pH water, resulted from the melting of snow [25]. One could speculate that the snowfall, which has an acidic character [25–29], might have affected the pH of the wastewater through “run off” phenomena. 

No other snowfall took place during the monitoring campaign, thus no definite conclusion can be drawn for a possible relationship between pH and snowfalls. 

### 3.2. Results for Chemical Oxygen Demand (COD)

**Figure 3:**
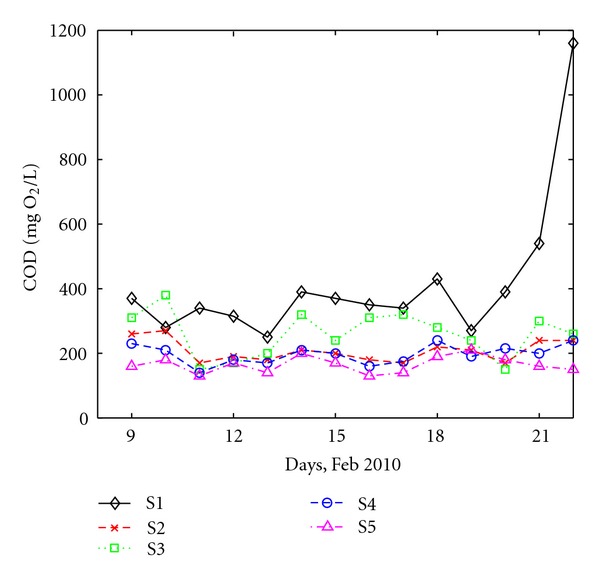
Daily variation of COD at all sites.

Detection of COD values in each sampling site of wastewater is presented in Figure 3. 

All COD values are higher than the maximum accepted values (125 mg O_2_/L) of the Romanian Law [24]. Both organic and inorganic compounds have an effect on urban wastewater's oxidability since COD represents not only oxidation of organic compounds, but also the oxidation of reductive inorganic compounds. That means some inorganic compounds interfere with COD determination through the consumption of Cr_2_O_7_^−2^ [30]. Two different behaviors can be observed, which are associated with the type of the collected wastewater as follows.

- *The first group *consists of stations S2, S4 and S5 where the wastewater has an important industrial component. At these stations, COD values are approximately between 150 and 300 mg O_2_/L, smaller, for instance, than COD values found by [31] in the raw wastewater produced by an industrial coffee plant where COD values were between 4000 and 4600 mg O_2_/L. Also, the temporal variation of COD values at all three stations is similar with no significant deviations from the average value, which is about 250 mg O_2_/L. Interestingly, the lowest COD level can be seen, on the average, at S5, which has the highest percentage of industrial wastewater.
- *The second group* comprises the “domestic” stations S1 and S3. The COD levels are higher, with values of 500 mg O_2_/L or more. Also, the variability is clearly higher than at the industrial-type stations. No clear association between the variations at the two sites can be seen. A peak in COD was measured in the 14th day of the study at site S1 (1160 mg O_2_/L). Since S1 is a domestic type station, it is unlikely that some major discharge led to such a high variation of COD. Unfortunately, no other information exists that might indicate a possible cause for this increase.

### 3.3. Results for Dissolved Oxygen (DO)

**Figure 4:**
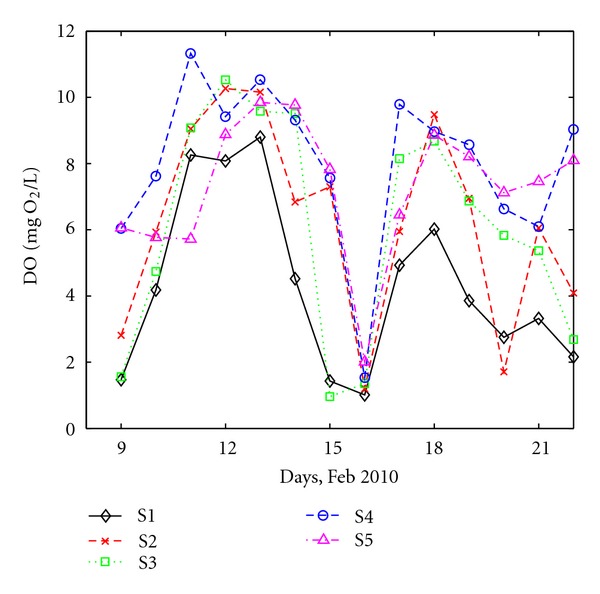
Daily variation of DO at all sites.

The amount of DO, which represents the concentration of chemical or biological compounds that can be oxidized and that might have pollution potential, can affect a sum of processes that include re-aeration, transport, photosynthesis, respiration, nitrification, and decay of organic matter [32]. Low DO concentrations can lead to impaired fish development and maturation, increased fish mortality, and underwater habitat degradation [33]. No standards are given by Romanian or European Law for DO in wastewater. The DO values for the analyzed wastewater at all five sites are shown in Figure 4.

Concentration of DO varies at all sampling sites and has values between 0.96 (at S2) and 11.33 (at S4) mg O_2_/L with a mean value of 6.39 mg O_2_/L. These are clearly higher than DO values measured, for instance, in surface natural waters in China, where the Taihu watershed had the lowest DO level (2.70 mg/L), while in other rivers DO varied from 3.14 to 3.36 mg O_2_/L [34]. On the other hand, such high values of DO (9.0 mg O_2_/L) could be found, for instance, in the Santa Cruz River [35], who argued that discharging industry and domestic wastewater induced serious organic pollution in rivers, since the decrease of DO was mainly caused by the decomposition of organic compounds. Extremely low DO content (DO < 2 mg O_2_/L) usually indicates the degradation of an aquatic system [34, 36]. 

The DO levels vary similarly for all selected sampling sites. The DO levels cover a wide range, with a minimum value of 1.0 mg O_2_/L at S1 and S3 and a maximum value of 11.33 mg O_2_/L at S4. There is a drop in DO at all stations, observed is in the 8th day of the monitoring interval, which coincides with the day when a similar decrease in pH took place. The lowest values of DO are observed for S1, one of the two “domestic” stations. It is interesting to note that DO at S5 is low although the wastewater here comes only from industry sources.

### 3.4. Metals

**Figure 5:**
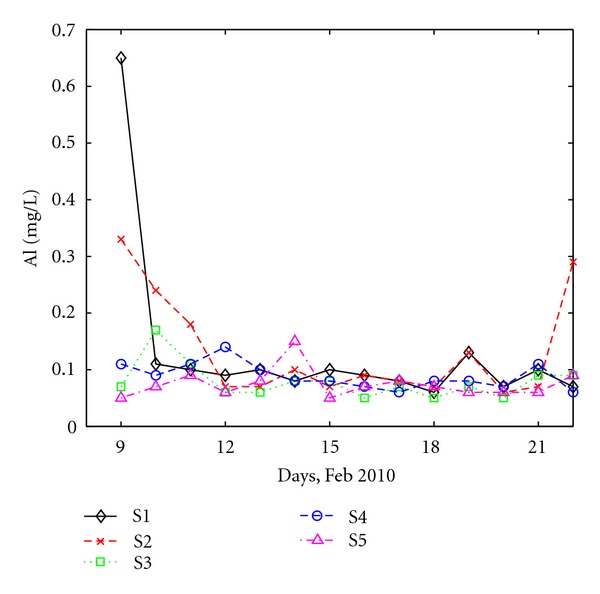
Daily variation of Al at all sites.

**Figure 6:**
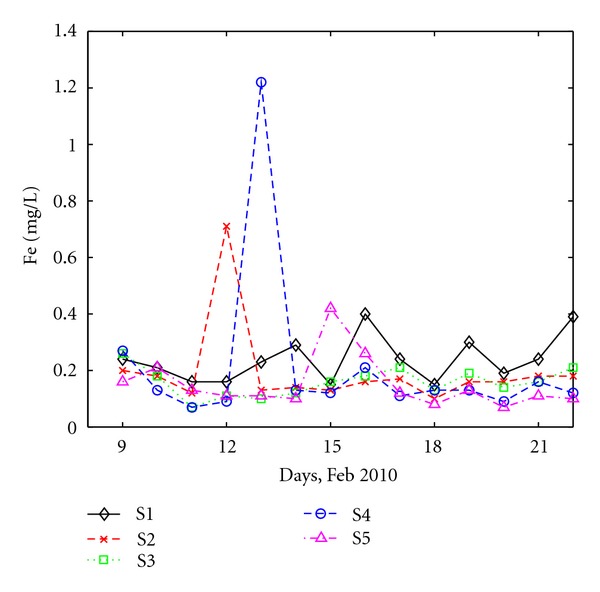
Daily variation of Fe at all sites.

**Figure 7:**
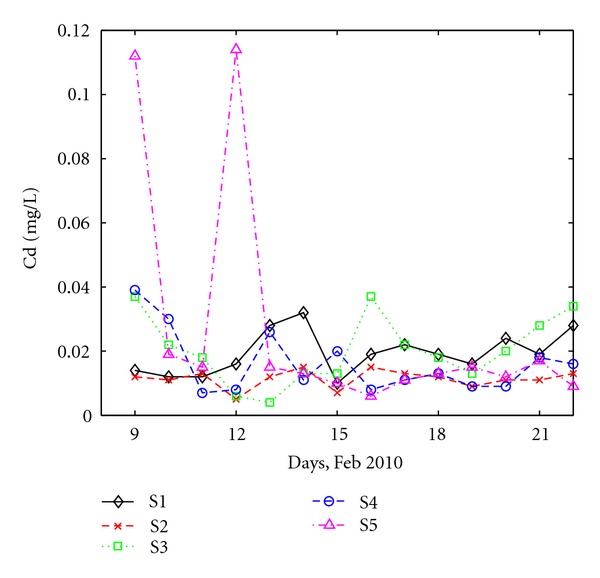
Daily variation of Cd at all sites.

The variation of Al^+3^, Fe^+2^, and Cd^+2^ concentrations in wastewater are shown in Figures 5, 6, and 7. Al^+3^ concentrations (Figure 5) were mostly within the 0.05–0.20 mg/L range at all the sampling sites. However, during the beginning and the end of the monitoring campaign, Al^+3^ concentration at station S2 is high (reaching even 0.65 mg/L), nonetheless below the limit imposed by the Romanian law, which is 5 mg/L [24]. The fact that in the beginning of the time interval, the concentration of Al^+3^ is high at two neighboring stations (S1 and S2) suggests that some localized discharge affecting both runaway and waste water, might have happened in the southern part of the city, which led to the increase of Al^+3^ concentration in the collected wastewater. This is supported by the fact that the concentration gradually decreases at S2.

The variation of Fe^+2^ concentrations is shown in Figure 6. Fe^+2^ concentration is within the 0.07–0.4 mg/L interval, below 5.0 mg/L, which is the maximum accepted value of the Romanian law [24]. Two higher values were observed at S2 and S4 (both with industrial component) during the third and fourth days of the monitoring campaign. 

Besides Al^+3^ and Fe^+2^, concentrations of Cd^+2^ were determined and the variations at the five stations are shown in Figure 7. Cd^+2^ is a rare pollutant, originating from heavy industry. Leakages in the sewage systems can also lead to Cd^+2^. Except for two days, Cd^+2^ varies between 0.005 and 0.04 mg/L. The two high values of 0.11 mg/L were observed in the first and fourth days at S5, which collects industrial wastewater. However, Cd^+2^ concentrations do not exceed the maximum accepted values of the Romanian law [24] for the monitoring interval which is 0.2 mg/L. 

### 3.5. Nutrients

**Figure 8:**
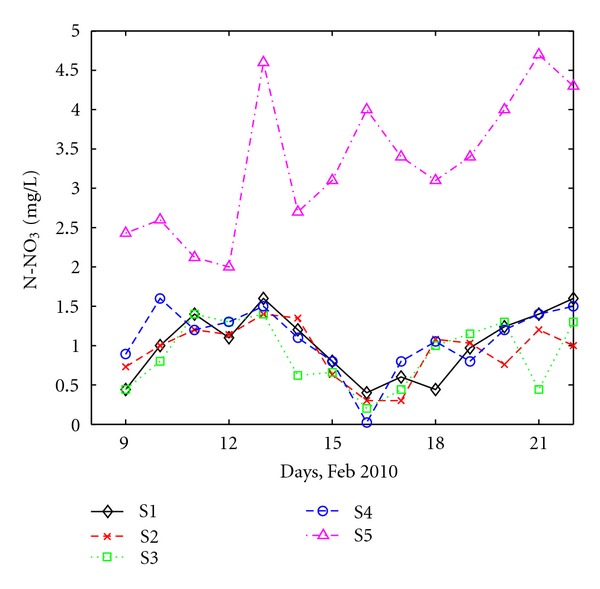
Daily variation of N-NO_3_ at all sites.

**Figure 9:**
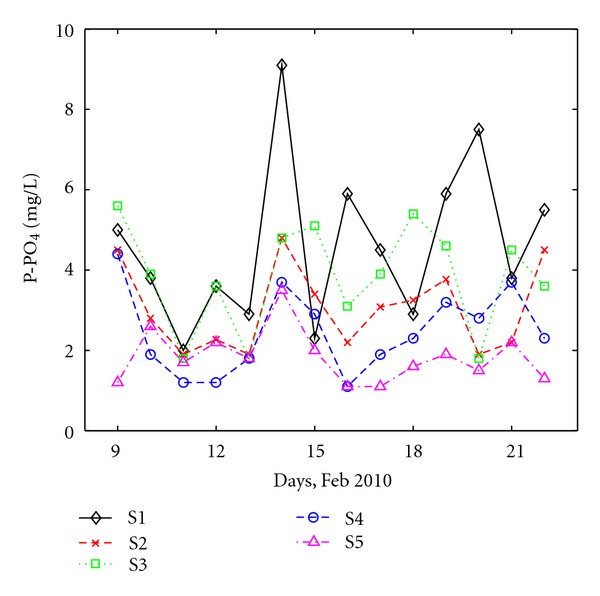
Daily variation of P-PO_4_ at all sites.

Water systems are very vulnerable to nitrate pollution sources like septic systems, animal waste, commercial fertilizers, and decaying organic matter [37]. Important quantities of nutrients, which are impossible to be removed naturally, can be found in rivers and this leads to the eutrophication of natural water (like Danube River). As a result, an increase in the lifetime of pathogenic microorganisms is expected. Measurement of nutrient (different forms of nitrogen (N) or phosphorous (P)) variations in domestic wastewater is strongly needed in order to maintain the water quality of receptors [36]. Nitrogen by nitrate (Figure 8) and phosphorous by phosphate (Figure 9) are considered as representative for nutrients.

Figure 8 shows that N-NO_3_ concentrations vary, on the average, between 0 and 5.0 mg/L.

At all four stations with a domestic component, S1, S2, S3 and S4, the concentration of N-NO_3_ is low (between 0 and 1.5 mg/L) and the daily variation is relatively similar at all sites. Noticeable drops of the N-NO_3_ concentration are observed at all stations in the 8th day of the monitoring interval, coinciding with pH (Figure 2) and DO strong variations (Figure 4). This supports the conclusion that the heavy snowfall recorded at that period had an important impact on wastewater quality most likely due to the runoff joining the sewage system.

The behavior of N-NO_3_ clearly differs at station S5, which collects only industrial wastewater. Significantly higher values of N-NO_3_, ranging from 2.0 to 5.0 mg/L, were detected. However, the mean concentration of N-NO_3_ remained below the maximum concentration given by the Romanian law [24]. Obviously, if treatment stations have to be set up, the priority for this particular nutrient component should concentrate on stations where industrial wastewater is collected. 

Another nutrient that was analyzed for our study was orthophosphate expressed by phosphorous. The P-PO_4_ concentration varies, on the average, between 1.0 and 6.0 mg/L (Figure 9). For this component, concentrations are higher at domestic stations, S1 and S3, than at the other three stations. P-PO_4_ is expected to increase in domestic wastewater because of food, more precisely meat, processing, washing, and so forth. The lowest values were observed at S5, which has a negligible domestic component. Peaks in the P-PO_4_ concentration are observed at S1. Interestingly enough, P-PO_4_ temporal variations correlated pretty well at stations S2, S4, and S5 (which collect industrial wastewater). Unlike most of the other analyzed compounds, for which the concentrations were within the accepted ranges, the maximum level of P-PO_4_ is exceeded at all five collectors. Both Romanian law [24] and the European law [23] stipulate 2.0 mg/L total phosphorous for 10000–100000 habitants, and for more than 100000 habitants (as in Galati City's case) 1.0 mg/L total phosphorus. Interestingly, domestic stations seem to require more attention with respect to the quality of water then industrial stations.

Our results regarding the variation and levels of the analyzed parameters are grouped below as the following.

1. The values of pH are within the accepted range for Danube [24], and their daily variations are relatively similar for both domestic and mixed wastewater. Significantly smaller pH values were measured in the wastewater with a high industrial load. A clear minimum was observed at all sites in the 8th day of the monitoring period, when a heavy snowfall took place. One could speculate that the snowfall, which has an acidic character [25–29], might have affected the pH of the wastewater through “run off” phenomena. However, a clear connection cannot be established relying on one event only.
2. The COD level clearly depends on the type of wastewater. Higher values were observed for domestic wastewater, while “pure” industrial wastewater has the lowest COD. This might be explained by the fact that industrial wastewater benefits from some treatment before being discharged into the city sewage system. However, COD does exceed the maximum accepted values according to the Romanian law [24] at all sites thus additional treatment is required at all stations.
3. Concentrations of all analysed metals, Al^+3^, Cd^+2^ and Fe^+2^, are within the limit of the Romanian law [24]. No association with the type of wastewater could be inferred. Isolated peaks could not be linked with any specific polluting factors, except for Cd^+2^, for which accidental concentration increases are observed for pure industrial wastewater.
4. The level of P-PO_4_, one of the two nutrients that were analyzed, was high at all stations; however, the highest concentrations are associated with domestic loads.
5. Opposingly, the N-NO_3_ level is the highest, by far, in wastewater with a high industrial contribution.

### 3.6. Possible Relationships between Various Parameters

The experimental results have shown that some parameters might be related and that their behavior greatly depends on the type of collected wastewater. Differences between the behavior of physico-chemical parameters at the domestic sites (S1 and S3), on one hand, and at the other sites, on the other, was observed. Pearson correlation coefficients have been calculated between all parameters at all the selected five sites and corresponding significances. Although most of correlations were not significant, some interesting connections between various parameters at sites with similar characteristics were revealed. Table 2 shows correlation coefficients between various parameters for all five stations. Significant correlations at different types of stations are denoted as follows: italicized fonts for domestic stations, boldface italicized fonts for the industrial station and boldface fonts for mixed stations.

An important relationship seems to exist between pH and N-NO_3_ at all stations except for the industrial wastewater collecting site, S5 (i.e., at all stations collecting wastewater resulting from domestic activities). Similarly, pH correlates well with DO at all stations except the industrial one. 

COD correlates with two metals, Cd^+2^ and soluble Fe^+2^, which is expected [30], but only at S1 and S3, where the daily variations of the concentration for these two metals (Cd^+2^ and soluble Fe^+2^) were similar. 

 No conclusion can be drawn for the industrial wastewater collector that was analyzed, where both positive and negative correlations were observed. The lack of correlation between the two metals and COD at the industrial wastewater collectors suggests that other processes, that alter the chemical equilibrium between the two chemical compounds, must be taken into account. For example some metals are complexed by organic compounds that are present in the water and the pH values can influence these phenomena.

DO correlates with pH and N-NO_3_ at all four sampling stations with domestic component (S1–S4) but the relationship vanish at S5 (industrial). There is also a negative correlation between DO and Fe^+2^ and Cd^+2^ only for domestic wastewater, which is expected because of the natural oxidation of metals. The correlation vanishes at the other three stations which collect wastewater from industrial areas. 

Heavy metals, Fe^+2^ and Cd^+2^ correlate only at domestic stations and no relationships can be defined to link the concentration of Al^+3^ with other components.

The P-PO_4_ variation is linked to the variation of soluble Fe^+2^ at the two stations that collect domestic wastewater. Interestingly, these two elements exist together in reductive domestic systems because these are dominated by proteins, lipids, degradation products. This relationship disappears at the other stations, where the industrial load is significant. The other metals, Al^+3^, seems to be linked with P-PO_4_ at stations S5 and S2, which collect wastewater with the highest industrial load. No link is observed for the rest of stations and for Cd^+2^ which can be explained by a higher probability of iron (II) orthophosphate to form in wastewater compared to Al^+3^ or Cd^+2^ orthophosphates. 

Positive correlations can also be seen between P-PO_4_ and COD for all sampling sites except S1, where the relationship is still positive but less significant. The other nutrient, N-NO_3_, is anticorrelated with COD but only at S3 and is well correlated with pH and DO at all four stations with domestic component. The only exception is station S5, which collects mostly industrial wastewater. 

Concluding, positive correlations were observed between the following parameters.

1. pH and N-NO_3_ everywhere except “purely” industrial water.
2. COD and soluble Fe^+2^ at domestic stations.
3. DO and pH, on the one hand, and DO and N-NO_3_ at domestic stations.
4. P-PO_4_ and soluble Fe^+2^ at domestic stations.
5. P-PO_4_ and COD everywhere, which, taking into account the high level of P-PO_4_ at domestic stations, might suggest that one important contributor to water quality degradation are household discharges.
6. Al^+3^ and P-PO_4_.

## 4. Conclusions

In the present paper we have analyzed the daily variation of several physico-chemical parameters of the wastewater (pH, COD, DO, Al^+3^, Fe^+2^, Cd^+2^, N-NO_3_, and P-PO_4_) at five collectors that have been characterized as domestic, industrial and mixed, according to the type of collecting area. Different results have been obtained for domestic and industrial wastewater. Most of the chemical parameters are within accepted ranges. Nevertheless, their values as well as their behavior depend significantly on the type of collected wastewater.

The overall conclusion is that wastewater with a high domestic load has the highest negative impact on water quality in a river. On the other hand, industrial wastewater brings an important nutrient load, with potentially negative effect on the basins where it is discharged. Our results suggested that meteorological factors (snow) might modify some characteristics of wastewater, but a clear connection cannot be established relying on one event only.

Significantly smaller pH values were measured in the wastewater with a high industrial load. The COD level clearly depends on the type of wastewater. Higher values were observed for wastewater with domestic sources, while “pure” industrial wastewater has the lowest COD. This might be explained by the fact that industrial wastewater benefits from some treatment before being discharged into the city sewage system. COD does exceed the maximum accepted values according to the Romanian law [24] at all sites thus additional treatment is required at all stations. Accidental increases of Cd^+2^ concentrations are observed for pure industrial wastewater. The highest concentrations of P-PO_4_ are associated with domestic loads. Opposing, the N-NO_3_ level is clearly the highest in wastewater with a high industrial contribution.

Correlation analysis has been used in order to identify possible relationships between various parameters for wastewater of similar origin.

Positive correlations between various physico-chemical parameters exist for the domestic wastewater (DO, pH and N-NO_3_, on the one hand, and P-PO_4, _COD and soluble Fe^+2^, on the other hand). Except for two cases, these relationships break when the industrial load is high. Some of the existing correlations are expected as discussed above, thus any removal treatment should be differentiated according to the type of collector, before discharging it into the natural receptors in order to be costly efficient. Correlations between DO and COD and nutrient load suggest that the most important threat for natural basins in the studied area, are domestic sources for the wastewater.

The different percentages of industrial and domestic collected wastewater vary at each station, which has a clear impact on concentrations of the selected chemical components. Our results show that domestic wastewater has a higher negative impact on water quality than wastewater with a high industrial load, which, surprisingly, seems to be cleaner. This might be related to the fact that most industries are forced, by law, to apply a pretreatment before discharging wastewater into the city sewage system. Industrial wastewater affects the nutrient content of natural water basins. Although the time period was relatively short, our study identified specific requirements of chemical treatment at each station. An efficient treatment plan should take into account the type of wastewater to be processed at each station. Results presented here are linked with another research topic assessing the level of water quality in the lower basin of the Danube before and after implementing the complete biochemical treatment plants. 

## Figures and Tables

**Table 1:** Characteristics of collectors S1,…, S5.

Collector/type of wastewater	Area covered by collector (ha)	Total length of pipes (m)	Percentage of the entire sewerage system (%)	Industrial (%)	Domestic (%)	Collector flow rate (m^3^/day)
S1/domestic	76	1775	**10**	4	96	11068
S2/mixed	1219	5728	**64**	70	30	62949
S3/domestic	59	1245	**5**	5	95	5206
S4/mixed	585	4552	**11**	30	70	14074
S5/industrial	345	4689	**10**	96	4	2699

**Table 2:** Correlation coefficients calculated for station S1 to S5. Significant correlations at each type of stations are identified as follows: boldface italicized fonts for industrial station (S5), italicized fonts for domestic stations (S1 and S3) and boldface fonts for mixed stations (S2 and S4).

Correlation factor		COD	pH	N-NO_3_	P-PO_4_	Al	Fe	Cd
	*S1*	0,21						
	**S2**	0,00						
pH	*S3*	−0,30						
	**S4**	−0,15						
	***S5***	0,41						

	*S1*	0,36	0,40					
	**S2**	0,20	**0,53**					
N-NO_3_	*S3*	*−0,76*	*0,61*					
	**S4**	0,28	**0,49**					
	***S5***	−0,22	−0,30					

	*S1*	0,14	0,12	0,01				
	**S2**	**0,52**	−0,06	0,00				
P-PO_4_	*S3*	*0,61*	−0,18	*−0,54*				
	**S4**	**0,60**	−0,42	0,05				
	***S5***	***0,57***	0,31	−0,28				

	*S1*	−0,11	−0,21	−0,37	0,03			
	**S2**	**0,66**	−0,26	0,00	**0,51**			
Al	*S3*	0,38	0,18	0,00	0,01			
	**S4**	−0,26	0,05	0,33	−0,04			
	***S5***	0,08	0,02	−0,09	***0,54***			

	*S1*	*0,48*	0,13	0,03	*0,55*	0,00		
	**S2**	−0,06	0,10	0,11	−0,16	−0,09		
Fe	*S3*	*0,60*	*−0,55*	*−0,54*	*0,52*	0,04		
	**S4**	−0,21	0,24	0,21	−0,10	0,15		
	***S5***	−0,13	−0,16	−0,12	−0,02	−0,33		

	*S1*	0,37	*0,67*	0,40	*0,61*	−0,29	*0,46*	
	**S2**	−0,02	**−0,49**	−0,10	0,18	0,21	**−0,64**	
Cd	*S3*	*0,48*	*−0,79*	*−0,55*	0,20	0,09	*0,69*	
	**S4**	0,39	−0,15	0,29	0,43	0,20	0,40	
	***S5***	0,03	−0,11	***−0,54***	−0,04	−0,32	−0,10	
